# Transmission, adaptation and geographical spread of the *Pseudomonas aeruginosa* Liverpool epidemic strain

**DOI:** 10.1099/mgen.0.000511

**Published:** 2021-03-15

**Authors:** Matthew P. Moore, Iain L. Lamont, David Williams, Steve Paterson, Irena Kukavica-Ibrulj, Nicholas P. Tucker, Dervla T. D. Kenna, Jane F. Turton, Julie Jeukens, Luca Freschi, Bryan A. Wee, Nicholas J. Loman, Stephen Holden, Susan Manzoor, Peter Hawkey, Kevin W. Southern, Martin J. Walshaw, Roger C. Levesque, Joanne L. Fothergill, Craig Winstanley

**Affiliations:** ^1^​ Institute of Infection and Global Health, University of Liverpool, Liverpool, UK; ^2^​ Department of Biochemistry, University of Otago, Dunedin, New Zealand; ^3^​ Institute of Integrative Biology, University of Liverpool, Liverpool, UK; ^4^​ Institute for Integrative and Systems Biology, Université Laval, Quebec City, QC, Canada; ^5^​ Strathclyde Institute of Pharmacy & Biomedical Sciences. University of Strathclyde, Glasgow, UK; ^6^​ National Infection Service, Public Health England, London, UK; ^7^​ School of Chemistry and Molecular Biosciences, The University of Queensland, Brisbane, QLD 4072, Australia; ^8^​ Institute for Microbiology & Infection, University of Birmingham, Birmingham, UK; ^9^​ Nottingham University Hospitals NHS Trust, Nottingham, UK; ^10^​ University Hospitals Birmingham, Heartlands Hospital, Bordesley Green East, Birmingham, UK; ^11^​ Alder Hey Children’s Hospital, Liverpool, UK; ^12^​ Liverpool Heart and Chest Hospital, Liverpool, UK; ^†^​ Present address: Nuffield Department of Health, University of Oxford, UK; ^‡^​ Present address: Harvard Medical School, Boston, MA, USA; ^§^​ Present address: Usher Institute, University of Edinburgh, Edinburgh, UK; ^#^​ Present address: MSD Research Laboratories, Two Pancras Square, London, UK; ^¶^​ Present address: University of Birmingham Microbiome Treatment Centre, College of Medical and Dental Sciences, University of Birmingham, Birmingham, UK

**Keywords:** cystic fibrosis, genomics, prophage, *Pseudomonas aeruginosa*

## Abstract

The Liverpool epidemic strain (LES) is an important transmissible clonal lineage of *Pseudomonas aeruginosa* that chronically infects the lungs of people with cystic fibrosis (CF). Previous studies have focused on the genomics of the LES in a limited number of isolates, mostly from one CF centre in the UK, and from studies highlighting identification of the LES in Canada. Here we significantly extend the current LES genome database by genome sequencing 91 isolates from multiple CF centres across the UK, and we describe the comparative genomics of this large collection of LES isolates from the UK and Canada. Phylogenetic analysis revealed that the 145 LES genomes analysed formed a distinct clonal lineage when compared with the wider *P. aeruginosa* population. Notably, the isolates formed two clades: one associated with isolates from Canada, and the other associated with UK isolates. Further analysis of the UK LES isolates revealed clustering by clinic geography. Where isolates clustered closely together, the association was often supported by clinical data linking isolates or patients. When compared with the earliest known isolate, LESB58 (from 1988), many UK LES isolates shared common loss-of-function mutations, such as in genes *gltR* and *fleR*. Other loss-of-function mutations identified in previous studies as common adaptations during CF chronic lung infections were also identified in multiple LES isolates. Analysis of the LES accessory genome (including genomic islands and prophages) revealed variations in the carriage of large genomic regions, with some evidence for shared genomic island/prophage complement according to clinic location. Our study reveals divergence and adaptation during the spread of the LES, within the UK and between continents.

## Data Summary

The GenBank accession numbers of the genomes used in this study that have previously been deposited in the National Center for Biotechnology Information (NCBI) database, along with the published studies they were analysed for, have been provided in Table S1 (available in the online version of this article). Genomes sequenced for this study have been deposited in the NCBI Genome database under the BioProject ID PRJNA615626 and their accession numbers are provided in Table S1.

**Impact Statement**

Chronic lung infections caused by *Pseudomonas aeruginosa* are the major cause of the morbidity and mortality associated with cystic fibrosis. In the UK and Canada, a particular lineage of *P. aeruginosa* (the Liverpool epidemic strain, or LES) possesses unusual characteristics: it can transmit between patients and is associated with greater morbidity. In the UK, the LES lineage is the most common lineage found amongst CF isolates. Using whole-genome sequencing and comparative genomics analyses, we have analysed 145 LES isolates from across the UK and Canada, providing the most comprehensive analysis to date of the LES lineage. We demonstrate geographical clustering of LES isolates, and describe how the lineage has adapted and diverged. In particular, we show divergence between isolates in Canada compared to the UK, and variations between isolates from different UK clinics. Our analysis has identified adaptations that might contribute to the success of this lineage as a pathogen in cystic fibrosis.

## Introduction

Chronic lung infections with the versatile pathogen *Pseudomonas aeruginosa* remain the major cause of morbidity and mortality associated with cystic fibrosis (CF). During the infection process in the CF lung, the bacterium undergoes characteristic adaptive changes [1], making it difficult or impossible to eradicate once established. Because *P. aeruginosa* can prosper in a wide variety of environmental and clinical niches, exposure to it is inevitable, and it is still a wide assumption that the majority of CF patients acquire their infections from an environmental source. However, it has become clear that specialist clones of *P. aeruginosa* capable of patient-to-patient transmission exist [2, 3], leading to over-representation amongst isolates from CF. The occurrence of such strains presents additional challenges in terms of patient care, but their identification does enable steps to be taken to minimize transmission amongst patients [4]. One of the best-studied transmissible clones is the Liverpool epidemic strain (LES), first discovered in a children’s CF unit in Liverpool [5], but since identified as the most common single clone amongst CF isolates from patients in the UK [6]. The LES lineage has also been identified in Canada [7, 8]. The LES has a number of important characteristics, including the ability to superinfect [9], increased production of some virulence factors [10, 11], an association with increased morbidity [12], increased resistance to antimicrobials [13] and the ability to cause infections beyond the CF host, including infections of the non-CF parents of a CF patient [14] and a patient’s pet cat [15]. The strain has also been reported as causing pleural empyema in a CF patient [16] and has been isolated from patients with primary ciliary dyskinesia and non-CF bronchiectasis (unpublished data).

Since the first *P. aeruginosa* genome was published in 2000 [17], a number of other complete *P. aeruginosa* genomes have been made available [18–24]. Whole-genome sequence data have also been used for analyses of within-clone variation for the two most abundant *P. aeruginosa* clones globally (PA14 and clone C) [25]. The complete genome of the earliest known LES isolate (LESB58 from 1988) was published in 2009 [26], revealing a number of genomic features, including five GIs GIs) and six prophages, some of which have been implicated in the competitiveness of the strain *in vivo* [26, 27], and five of which can be detected as free phages in CF patient sputa [28]. Since then, 3 further genomes from LES isolates from Liverpool [29] and 12 from Ontario, Canada [8] have been reported. In addition, further LES isolate genomes have been sequenced from the adult CF unit in Liverpool in studies, suggesting the co-existence within patients of distinct LES sub-lineages [30–32].

The aim of this study was to determine the genome sequences of a larger collection of LES isolates from multiple centres throughout the UK and analyse the resulting extended dataset in order to characterize the diversity of a major CF transmissible strain in the context of geographical and temporal dissemination. Our data support the sub-division of LES isolates into distinct UK and Canadian clades, with some additional clustering by centre within the UK, and reveal mutational signatures of on-going adaptation to the CF lung environment.

## Methods

### Bacterial strains

Details of all the LES isolates included in the genomic analysis are given in Table S1. In total, 145 LES genomes were analysed, including the genomes of 91 isolates sequenced for this study. The majority of the newly sequenced isolates were obtained from the collection at Public Health England (PHE), Colindale or the archived collection in Liverpool. Additional UK genomes were obtained from CF centres in the West Midlands and East Midlands. Amongst the published genomes included were representatives of different sub-lineages of the LES identified in a study of isolates in the Liverpool Adult CF Unit [30].

### Genome sequencing of bacterial isolates

DNA was extracted from 91 LES isolates using the Promega Wizard genomic DNA extraction kit. Whole genomes were sequenced either by the Centre of Genomic Research (CGR), Liverpool, using Nextera library preparation and an Illumina HiSeq sequencer or at Institut de Biologie Intégrative et des Systèmes (IBIS), Université Laval, Canada on the Illumina MiSeq platform. An additional 54 previously published LES genomes were included in the analysis [8, 29, 30]. These included a resequenced strain LESB58, labelled as genome LESB58_2. Three hundred and ninety non-LES *P. aeruginosa* genomes were obtained from a published *P. aeruginosa* genomics study [23] to represent a wider *P. aeruginosa* population set.

### Genome assemblies

Short reads were adapter-trimmed and quality-filtered using cutadapt-1.9.2 [33] and assembled to the contig level using the A5 assembly pipeline [34]. The *de novo*-assembled contigs were scaffolded to the genome of LESB58 [26] using ABACAS [35]. Contigs containing sequence accessory to the LESB58 genome were appended to the end of each draft genome. All genomes were annotated with Prokka-1.12, including CRISPR prediction [36]. The pseudomonas.com database [37] was used to facilitate further assessment of gene function and the impact of mutations. Multilocus sequence types (MLSTs) were determined using mlst (https://github.com/tseemann/mlst) and the pubmlst database [38].

### Core genome phylogeny

In order to assess the placement of the LES lineage in the wider *P. aeruginosa* population, a relaxed core *P. aeruginosa* tree was inferred from the LES genomes and the 390 *P *. *aeruginosa* genomes from a previous genomics study [23]. LESB58 was designated as the initial reference genome for Panseq [39], followed by random successive reference selection for genomic regions not present in previous reference genomes. Successive reference genomes were split into 500 bp fragments and pairwise aligned to all other genomes. Fragments with at least 90 % blast percentage identity to the reference in 95 % of taxa (508/535) were designated as belonging to the relaxed core, aligned with clustalw [40] and concatenated with SCaFoS [41]. The resulting 5 517 752 bp supermatrix contained 429 463 informative sites. The tree was inferred by maximum likelihood (ML) with the HKY85 substitution model and 1000 ultrafast bootstrap (UFBoot) [42] replicates for branch support using IQ-TREE-1.6.2 [43]. To assess the root position of the LES lineage, this was repeated with the 145 LES genomes and *P. aeruginosa* reference genomes and known outgroups: PAO1 and PA14 [17, 44]. Fragments were defined as core if they had 90 % blast percentage identity with the reference and were present in 95 % of taxa (141/147). This resulted in a 5 840 172 bp relaxed core supermatrix including 14 730 informative sites. The TIM+R5 substitution model was determined as the best fit for the data by IQ-TREE [43] model test [45] and the phylogenetic tree was inferred by ML with 1000 UFBoot replicates, where at least 95 % confidence is recommended as well supported. The tree was rooted between outgroups PAO1 and PA14 and the rest of the tree. Finally, snippy (https://github.com/tseemann/snippy) was used with default parameters to generate a substitutions-only LES core single-nucleotide polymorphism (SNP) alignment from all LES assemblies and the tree was approximated using runListCompare (https://github.com/davideyre/runListCompare), which uses IQ-TREE [43] for model selection and phylogeny inference, and ClonalFrameML [46] for the identification of recombinant sites to be removed from the alignment. LESB58 GI and prophage regions [26] were also masked. The GTR+F+G4 substitution model was selected by IQ-TREE and there were 2947 informative sites. Phylogenetic trees were visualized with either the interactive tree of life software (iTol) [47] or GrapeTree [48]. SNP distances were plotted using the Python packages seaborn (https://seaborn.pydata.org/), matplotlib [49], pandas [50] and numpy [51] with the Jupyter Notebook environment [52].

### Core and accessory genes

The core and accessory gene content of the LES genomes was determined by Roary [53] using Prokka-annotated gff files as input and without splitting paralogues. This was repeated for the LES plus the wider *P. aeruginosa* population genomes, but with paralogue splitting. Genes determined to be ‘strict core’ in the pangenome analysis by Roary were present in >=99 % of the total set and relaxed core >=95 %. Association tests of accessory gene content and Benjamimi–Hochberg (BH) multiple testing corrections were performed by SCOARY [54] using the Roary-generated gene presence/absence matrix output as input. Abricate (https://github.com/tseeman/abricate) was used to investigate the potential presence of antibiotic resistance-conferring genes.

### Structural analysis

The assembled genomes were also compared to LESB58 for large, structural differences using the blast Ring Image Generator (BRIG) [55] to identify large (10 kb or greater) deletions. The presence and absence of virulence genes in the assembled genomes was investigated using Blastable v0.4, a Python script (https://github.com/bawee/blastable) that performs a nucleotide blast+ search. To further investigate the presence and absence of LESB58 prophages and GIs, a database of LES genomes was generated using blast+ [56] makeblastdb, against which each LESB58 prophage and GI was aligned using blast+ blastn (window size 28). Percentage identity was corrected for the contribution of duplicate regions present elsewhere in the draft genome by reciprocally aligning known duplicate regions with blastn [28].

### Identification of SNPs and microindels in UK LES relative to the genome of LESB58

High-quality SNPs and microindels in the UK LES genomes for which short reads were available were called against the completed LESB58 reference genome, which is the outgroup to the UK LES and the oldest dated LES isolate [26, 30]. Quality-filtered short reads were mapped to reference genome LESB58 with bwa0.7.5a mem [57]. The LESB58 genome (.*fasta*) was indexed with bwa-0.7.5a index [57] and samtools-0.1.18-r580 [58] faidx. A sequence dictionary was created with picard-tools-1.135 [59] –CreateSequenceDictionary. The resulting sequence alignment map (.*sam*) file from read mapping with bwa was converted to a binary alignment map (.*bam*) file using picard-tools SortSam. Duplicates were marked using picard-tools MarkDuplicates and a bam file index created with picard-tools BuildBamIndex. The Genome Analysis Toolkit-3.4 (GATK) [60] Realignor Target Creator was used to designate targets for indel realignment and indels were realigned with GATK IndelRealigner. Variants were called using GATK HaplotypeCaller (-ploidy 1-emitRefConfidence GVCF) to produce a variant call (.*vcf*) file prepared for genotyping. The vcf file was genotyped using GATK GenotypeGVCFs and filtered using vcffilter [61] basic filtering (DP >9 and QUAL >10). Variant annotation was performed by snpEff-4.1l [62] with the default parameters for GATK output (-eff -gatk) to the LESB58 reference genome annotation database, uid59275. Additionally, we determined whether genes had larger deletions not detectable due to lack of sequencing reads and absence of genomic context in vcf files. First bam files were indexed with samtools index and the reads aligned to a regions determined by snpEff database gene regions using samtools depth. Genes with any deletions larger than 20 bp were not considered for parsimonious assessment of convergent adaptation by loss of function.

## Results

### Genome assemblies

A geographically and historically diverse sample of LES isolates was selected for this study (*n*=91) and was combined with a set of previously sequenced LES genomes (*n*=54). The newly sequenced genomes were primarily isolated from CF patients, but also included some isolates from primary ciliary dyskinesia (PCD) patients, a pet cat (OSIRIS) [63], non-CF bronchiectasis (nCFBr) patients, a blood isolate (PHELES19) and the non-CF parents of a CF patient [14]. All LES genomes analysed in this study, and their geographical source (according to PHE regions) and date of isolation, if known, are presented in Table S1. The mean number of CDS per genome was 6004, with a range of 5854 (PHELES12) to 6506 (JD324). Nearly all LES genomes, with the exception of those from isolates prefixed with ‘JD’, and ‘MdLES’, were predicted to carry type 1 CRISPR sequences.

### The LES genomes form a distinct lineage amongst the wider *P. aeruginosa* population

**Fig. 1.**
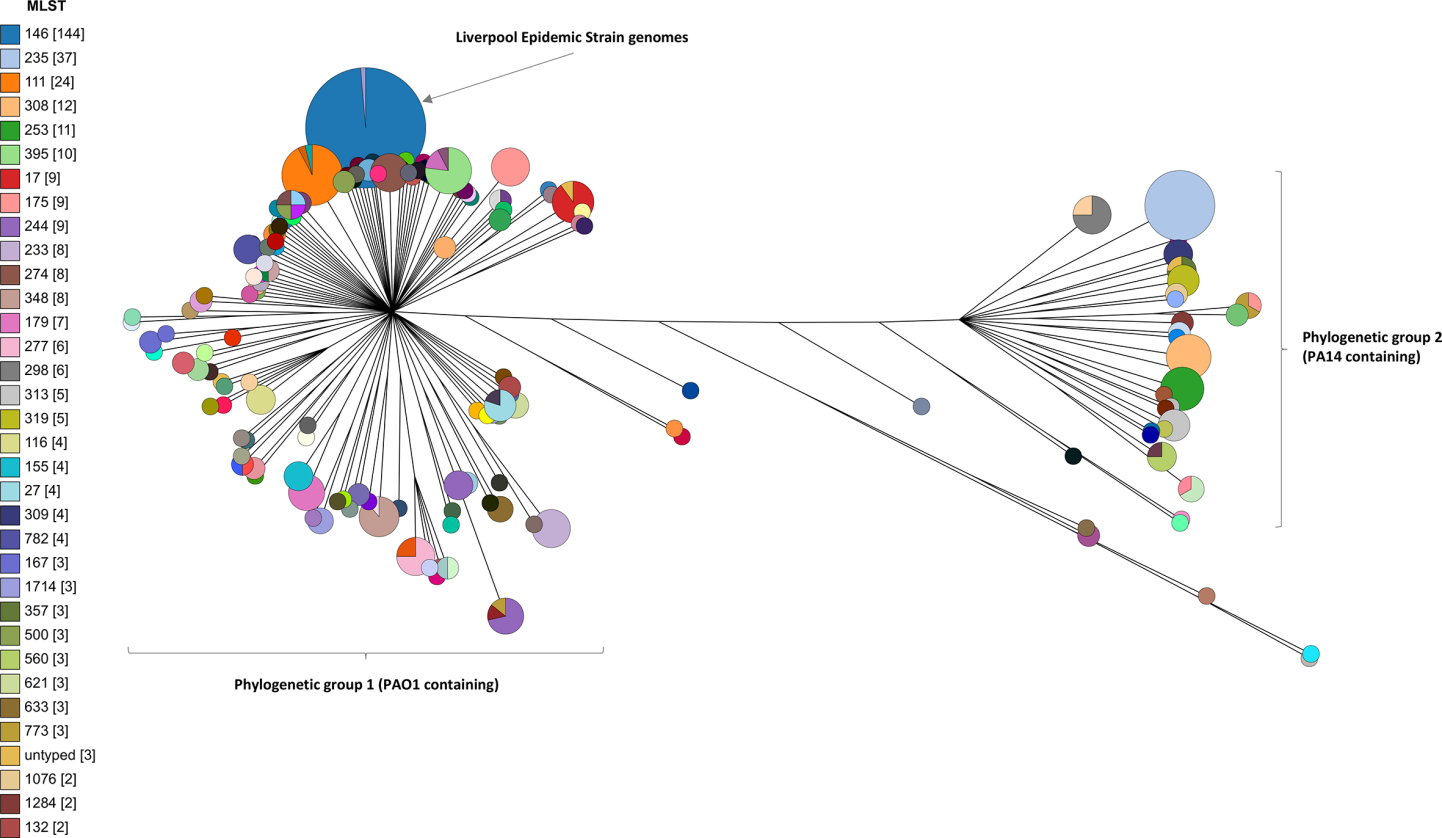
Phylogenetic inference of LES genomes and 390 genomes from a previous study representative of the wider *Pseudomonas aeruginosa* population. The tree was constructed with IQ-TREE [43] maximum likelihood, the HKY85 substitution model and 1000 ultra-fast bootstrap support replicates from a relaxed core fragment supermatrix (500 bp fragments present in >=95% of genomes) with 429463 informative sites. The tree was visualized by GrapeTree [48] with branches log-transformed and nodes collapsed and coloured by MLST. MLSTs are included in the key for those represented by two or more genomes

A relaxed core genome was extracted from a combination of all the LES genomes plus the wider *P. aeruginosa* population set. The LES was resolved as a monophyletic, clonal lineage (Fig. 1). The lineage is composed of two MLST sequence types, ST-146 (primarily) and ST-683, that differ in the *ppsA* locus. Of the 145 LES genomes, 128 were ST-146, 2 were ST-683 and 15 did not correspond to known STs, differing from ST-146 by 1 locus in each case. The wider tree largely resolved into two major *P. aeruginosa* phylogenetic groups, but with a few lineages being placed in neither of the major groups, consistent with previous analysis [64]. One genome from the wider population set was placed within the LES clade, namely isolate AZPAE13757_2337, associated with a respiratory tract infection (RTI) from Canada. This isolate also carries the LES serotype (06) and MLST (ST-146) and was therefore coded as LES in the gene association analysis [23].

A tree of the LES genomes included in this study, with additional genomes from the reference strains PAO1 and PA14, was reconstructed based on a relaxed core genome fragments supermatrix. The LES genomes clustered into two major phylogenetic groups, with clade I containing mainly Canadian isolates and clade II containing entirely UK-sourced isolates. The root position was determined with 100% UFBoot support to be within clade I (Fig. S1).

### Phylogenetic analysis reveals clustering by clinic geography

**Fig. 2.**
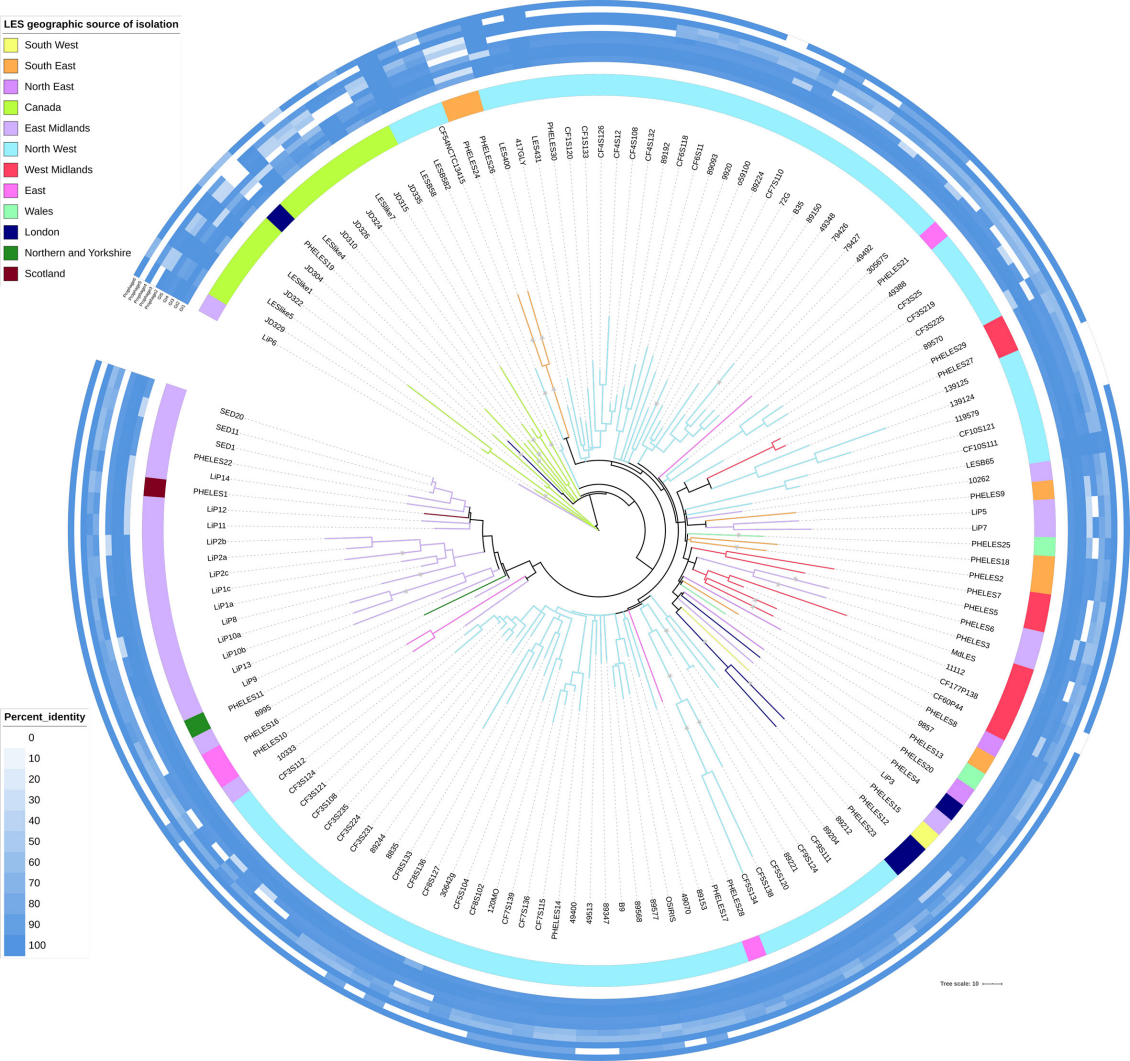
Phylogenetic tree inferred from core SNPs with LESB58 GIs and prophages masked. Recombinant sites determined by ClonalFrameML were excluded. The GTR+F+G4 substitution model was selected by IQ-TREE and there were 2947 informative sites. The tree was visualized by iTol [47] with colour strip and branch colours indicating PHE designated regions of isolation or Canada. For visualization branch lengths >70 SNPs were truncated and marked with a star. Plotted around the tree is a heatmap of percentage identity with LESB58 prophage and GI regions, prophages 2–6 and GIs 1–5.

The LES reference genomes representing the oldest known LES isolate, LESB58 (whole genome) and LESB58_2 (resequenced with Illumina data, 2.93 SNPs different) were placed between the two clades, and thus represented an outgroup to the rest of the UK genomes (Fig. 2). There were two main exceptions to the general rule of separation according to country of origin. Clade I contained two LES isolate genomes sampled from the UK: LiP6 and PHELES19. Further investigation revealed that the patient infected with LiP6 had lived in Canada, but no further information was available for the patient infected with PHELES19, other than the fact that this was a blood isolate. Within clade II there was phylogenetic evidence for further clustering by source of isolation within the UK and between patients known to be in contact with one another. Where genomes from one region or clinic were placed within a UK sub-clade dominated by genomes from a different clinic or region, further information was sought about possible previous patient links to the region. PHELES10 and PHELES16 were isolated from patients in the East of England in 2014, but fall within a majority East Midlands clade. Both patients were recorded as attending and presenting samples prior to 2014 in the East Midlands CF unit. PHELES1 was isolated from a patient in Scotland, but falls within a majority East Midlands clade; this patient had previously attended the East Midlands CF clinic. Although many sub-clades of the LES had a clear geographical association (Fig. 2), there were some examples of isolates from different origins clustering. For example, isolates PHELES20, 4, 15, 12 and 23 cluster together despite being from different geographical locations. However, we do not have sufficient information about patient histories to explain this.

The LES tree was corrected for recombination after likely recombinant regions were detected in 30 inner nodes of the tree and 109 tip-branches (Fig. 2). Within clade I the median recombination corrected pairwise branch length (193.92 SNPs) was longer than within clade II (119.72 SNPs), including LESB58 (Figs S2 and S3a). The longer clade I branch lengths could either be due to the lineages being older, sparse sampling or that the clade II genomes by comparison have transmitted faster. Overall, the median branch lengths within the LES tips were 131.11 SNPs (of 10 440 exclusive pairs).

### Phylogenetic analysis reveals clustering supported by clinical history

Individual CF patients (identified as isolates that are designated with names commencing with ‘CF’ followed by a common number, for example CF5S120, CF5S138 and CF5S134, all from patient CF5) typically clustered together. In a previous study by Williams *et al.* [30] with extensive within-patient sampling within a single CF clinic it was possible to determine that there were coexisting distinct lineages of the LES within individual patients. The coexistence of divergent lineages within patient CF3 were further confirmed here with two separate clusters (one containing CF3S124, CF3S235, CF3S224 and CF3S108; the other containing CF3S25, CF3S219 and CF3S225). The branch length between randomly selected genomes from each cluster, CF3S235 and CF3S219 was 108 SNPs.

The shortest branch lengths were most often between within-patient samples, though some branch lengths for isolates from different patients were shorter than between within-patient isolates, suggesting recent transmission or conversely distinct coexisting lineages within patients (Table S2). The shortest branch lengths from within-patient samples were SED11/SED20 SED1/SED11, SED1/SED20 [32, 65], with 0, 3.9 and 3.9 SNPs respectively and CF9S124/CF9S111, with 0.97 SNPs. Conversely, between-patient isolate genomes also had smaller branch lengths than some within-patient lineage branch lengths. These included 49492/30567S with 21.41 SNPs, 72G/B35 with 18.48 SNPs and PHELES27/PHELES29 with 10.69 SNPs.

There were a number of examples where isolates clustering together were linked based on clinical data. For example, isolates 139124 and 139125 (103.2 SNPs) from a single PCD patient, and isolate 119579 from their sibling (81.78 SNPs to 139 125), also a PCD patient, were adjacent on the tree. Isolates PHELES27 and PHELES29 (10.7 SNPs), and isolates 89204 and 89212 (82.7 SNPs), were isolated from siblings. Isolate CFS120 was from a CF patient and clustered with 417GLY (71.1 SNPs), isolated from their non-CF parent [63]. The isolate OSIRIS, isolated from a pet cat, clustered with the human isolate 89568 (28.21 SNPs), isolated from the owner. Isolates 79426 and 79427(166.6 SNPs), from the same empyema patient, clustered together.

Although many sub-clades of the LES had a clear geographical association (Fig. 2), there were some examples of isolates from different origins clustering. For example, isolates PHELES20, 4, 15, 12 and 23 cluster together, despite being from different geographical locations. However, we do not have sufficient information about patient histories to explain this.

### The LES accessory genome

The gene content for all LES genomes in this study (the LES pangenome) was compared with the pangenome of the wider *P. aeruginosa* population set. To assess significant associations between genes and designated groups of either the LES or the wider population set, Fisher’s exact test (<0.05) was used and BH correction for multiple testing [54]. A pangenome with 535 genomes, including the 145 LES genomes, was constructed. There were 3251 strict core genes (present in >=99 % genomes) of a pangenome of 33 033 different genes (where paralogous groups within the same genome were classified as the same gene). Compared with the wider population set, there were 2922 genes significantly associated with the LES by absence and 1397 genes significantly associated with the LES by presence. There were 152 genes present in >=95 % of the LES genomes but <=10 % of the wider population. Of these 108 are located within LESB58 GIs or prophages, particularly LES GI-3 (48 genes), LES prophage 3 (25 genes), LES prophage 4 (17 genes) and LES GI-1 (15 genes) (Table S3). There were 22 genes that were present in >=95 % of the wider population and <=10 % of the LES (Table S4).

We conducted further analyses to identify large regions that were absent from some LES isolate genomes. We interrogated the LES genomes using abricate in order to seek evidence for horizontal acquisition of antibiotic resistance genes. In addition to identifying resistance-related genes common to all isolates, there was evidence for additional beta-lactamase genes in several isolates. In particular, isolates from three different CF patients (CF3S108, CF4S132 and CF7S115) harboured class A beta-lactamase TEM-181 genes sharing 100 % identity with a gene in *Escherichia coli* (accession number NG_050218.1). This gene was not carried by other LES isolates from the same patient sputum samples.

Deletions of more than 10 kb were present in 24 of the LES sequenced genomes (Fig. S4, Table S5). Twenty eight deletions were identified, with seven of the deletions being greater than 100 kb, and the largest being 249 kb. CF9S111 and CF9S124 contained the same deletion, consistent with their high sequence similarity and adjoining positions on the phylogenetic tree (Fig. 2); the same is true of JD304 and LESlike1. Most strains only contained one deletion, although four strains contained two. The deletions were non-randomly distributed in the genome, with 15 of the 24 strains having deletions in the region between 3.10 and 3.50 Mb (Fig. S4). This region of the LESB58 genome contains, amongst others, genes encoding an extracellular polysaccharide (Psl), a siderophore (pyoverdine) and a type VI secretion system (Table S6).

There were also variations in the carriage of previously reported [26] LES GIs and prophage regions (Fig. 2). LES prophage 5 was more commonly absent from the LES isolate genomes. Carriage of prophage 2 and GI5 was also highly variable, with some evidence for shared GI/prophage complement according to geographical regions (Fig. 2). For example, the absence of GI5 was a feature of isolates from the East Midlands region. Likewise, there is a cluster of isolates from the Liverpool region that lack LES prophages 2 and 5. All of the isolates from Canada lacked LES prophage 5, but there were variations with respect to both carriage and divergence for other GI and prophage regions (Fig. 2).

### Mutations in LES genomes

Genomes of clade II (entirely UK-sourced) isolates for which short reads were available for high-quality read mapping and variant calling (*n*=124) were assessed for mutations compared with the oldest known LES isolate LESB58 (from 1988), as reference and outgroup to the clade II LES. There were 19 679 variants counted across all UK LES compared with LESB58, including 10 036 different mutations in 3742 different genes. The majority of variants by mutational impact category were amino acid substitutions (54.94 %), followed by synonymous nucleotide substitutions (20.24 %), frame-shifting mutations (9.96 %), mutations upstream of coding regions (9.56 %) and premature stop codons gained (2.60 %). The remainder (2.69 %) comprised codon insertions, codon deletions, codon changes plus insertions, mutations downstream of coding regions, stop codon loss, start codon loss, synonymous stop codon substitution and non-synonymous start codon substitutions. The majority of different mutations (7507/10 036) were only present in one genome. One hundred and six of the different mutations were present in 10 or more genomes, including 34 carried in each of 100 or more genomes. Mutations in or upstream of 20 different genes were found in all 124 UK LES genomes relative to LESB58 (Table S7). Two of these were synonymous mutations (in *phuR* and *wbpY*)*,* and two were located upstream of genes. The remaining 16 were amino acid substitutions in genes, including in the *nosR* gene, encoding a regulatory protein. It is possible that in these cases, LESB58 is the isolate that has acquired a mutation. However, the genome of LESB58 is identical to strain PAO1 in the region of the *nosR* mutation, and the mutation identified is not present in any of the Canadian LES, suggesting that it is a genuine mutation relative to LESB58 in all the UK LES isolates.

The reported 14 bp deletion in *gltR*, previously believed to be fixed in UK LES genomes other than LESB58 [29], was present in 100/124 UK LES genomes. Of the 24 UK isolates not harbouring this deletion, all but one (CF54NCTC13415) were associated with a majority East Midlands cluster (from isolate 10333 to isolate SED1 on the tree in Fig. 2). This mutation is not present in any of the Canadian LES. A nonsense mutation in *fleR*, also not found amongst the Canadian isolates, was present in 122/124 UK LES genomes (Table S7). An additional isolate (8835) carried a 28 bp deletion in *fleR* spanning the same locus of the nonsense mutation. LiP1a was the only isolate genome analysed without a nonsense mutation in *fleR*. However, the closely related LiP1c, isolated from the same patient as LiP1a, did harbour the nonsense mutation in *fleR* (Fig. 2, Table S7).

There were more non-synonymous mutations in some genes than others, suggesting ongoing adaptation as the strain spread around the UK. The majority of different genes carrying a nonsense mutation represented a single acquisition (615/742), including those present in only one isolate genome. The remaining 127 different genes were inferred to have independently acquired nonsense mutations in the population between 2 and 32 times. Those with four or more independent acquisitions in the population are displayed in Table 1, and include genes associated with functions such as antibiotic resistance, quorum sensing, mucoidy, iron acquisition, amino acid metabolism, chemotaxis, cell wall/LPS/capsule synthesis and central metabolism (Table 1). The porin protein-encoding gene, *oprD,* had independently acquired nonsense mutations more than any other gene in the population, acquiring nonsense mutations a minimum of 32 times and carried by 51/124 genomes. A further six genomes contained larger deletions ranging from 141 to 647 bp (10.63–48.79 %) within the 1326 bp *oprD* gene, bringing the total number of UK LES genomes with strong genetic evidence of loss of function in OprD to 57. MexA and MexB multidrug efflux pump proteins were both inferred to be adaptive by loss of function (Table 1) and are both encoded by the *mexAB-oprM* operon. Taken together, the genes encoding the MexAB-OprM efflux pump independently acquired loss-of-function mutations at least 26 times, in 50 isolates.

**Table 1.** LESB58 genes in the UK LES population inferred to have independently acquired nonsense mutations four or more times

No. of independent acquisitions	Gene name	No. of genomes	Gene description
**32**	*oprD*	51	Imipenem outer-membrane porin
**18**	*lasR*	26	Transcriptional regulator of quorum sensing
**21**	*mexB*	43	Multidrug efflux pump transporter, part of the *mexAB-oprM* operon
**20**	PLES_59241	32	Hypothetical membrane protein
**13**	*mucA*	29	Anti-sigma factor for alginate biosynthesis
**10**	*glpT*	15	Sn-glycerol-3-phosphate transporter
**9**	*mltD*	55	Murein transglycosylase D precursor
**8**	*pncA*	10	Nicotinamidase
**7**	PLES_34011	13	TonB-dependent receptor
**6**	PLES_48481	16	Hypothetical protein
**6**	*pmrB*	6	Two-component regulator system
**5**	PLES_46251	12	Putative glycosyl transferase
**5**	*opmQ*	7	Putative outer-membrane protein precursor
**5**	PLES_16021	5	Transcriptional regulator (*marR* orthologue, involved in repression of MDR mar operon)
**5**	*mpl*	11	UDP-N-acetylmuramate:L-alanyl-gamma-d-glutamyl-meso-diaminopimelate ligase
**5**	*chpA*	5	Chemosensory system component
**5**	PLES_51581	5	Putative S-adenosylmethionine decaboxylase proenzyme
**5**	*mdoH*	13	Glucosyltransferase involved in biofilm formation
**5**	*ampD*	111	N-acetyl-anhydromuranmyl-l-alanine amidase
**4**	PLES_46401	101	Hypothetical protein with filamentous haemagglutinin outer-membrane domain)
**4**	PLES_03151	9	Transcriptional regulator
**4**	PLES_47771	8	Two-component sensor
**4**	*pchE*	7	Dihydroaeruginoic acid synthase
**4**	*mexA*	7	Multidrug efflux pump transporter, part of the *mexAB-oprM* operon
**4**	PLES_07571	5	Putative oxidoreductase
**4**	PLES_29121	7	Putative ATP-binding/permease fusion ABC transporter involved in sulphate metabolism

Deficiency in DNA mismatch repair as a result of disruption to genes *mutS, mutL, mutM* and *uvrD* can result in the hypermutable phenotype, another adaptation that has been observed previously amongst *P. aeruginosa* CF isolates [66]. In the LES dataset, 10/124 genomes investigated were identified as probable hypermutators due to DNA mismatch repair deficiency. There was also evidence of cross-infection of hypermutable lineages. Isolates LiP10a/b and LiP13, from two different CF patients attending the same centre, are adjacent on the core SNP tree (Fig. 2) and carry a 4 bp deletion in *mutM* at the same position. In addition, isolates PHELES3 and PHELES6, also from to different patients attending the same CF centre, are adjacent on the core SNP tree (Fig. 2) and share 57 bp of a 58 bp deletion in *mutM*.

## Discussion

### Phylogenetic analysis of the LES

Previously, a limited number of LES genomes were available for comparative genomics [8, 29] and studies focused on the within-host diversity and adaptation of LES lineages during chronic lung infections [30, 65]. As well as greatly increasing the sample size for comparative genomics, the panel of LES isolates for this study significantly extends the geographical diversity of samples, and the diversity of associated pathologies. Using this extended dataset, we show that the LES forms a distinct clonal lineage, and that UK and Canadian LES isolates form distinct clades (Figs 1 and 2). There were two exceptions in the 145 isolate dataset, both of which were sourced in the UK but clustered with the isolates from Canada. For one (LiP6), further clinical data linked the patient with Canada. We were unable to find sufficient information to explain the location of the second (PHELES19). Nevertheless, the divergence between the isolates from Canada and UK, with the earliest known LES isolate (LESB58) lying between the groups, suggests that the LES may have established itself in both countries some decades ago and then undergone within-country spread.

There were seven genes with mutations leading to amino acid substitutions in all of the clade II genomes (UK) but none of the clade I genomes (Canada), and a further gene PLES_53461 with a mutation upstream in all clade II genomes. These included genes encoding the regulatory protein NosR, a predicted two-component regulatory protein (PLES_28161), transport proteins (PLES_13941 and PLES_44581), a ring cleavage dioxygenase (PLES_44361), a putative hydroxylase (PLES_48951) and DNA gyrase (*gyrB*). loss-of-function mutations in genes encoding two other regulatory proteins (FleR and GltR) were also found in the majority of clade II isolates. It is possible that some of these mutations have contributed to the adaptations allowing this strain to be transmissible. However, if this is the case, then the spread of the clade I isolates in Canada must have been facilitated by other adaptations.

There was evidence for clustering by geography within the UK. Again, there were exceptions, some of which could be explained by follow-up epidemiological investigations. Our sample was dominated by isolates from the North West (Liverpool) region. Notably, not all isolates from this region clustered together. Rather, there were several separate clusters, some indicating closer relationships with isolates from other regions, consistent with some sub-lineages seeding geographical spread, followed by divergence. It is not uncommon for CF patients to move from one area to another within the UK, and we cannot construct an entirely accurate natural history of the strain based on the limited clinical and epidemiological data available.

### Variations in the accessory genome content

The genome of the earliest isolate of the LES (LESB58) contains large GIs and prophages, some of which harbour genes shown to contribute to competitiveness in an animal model of chronic infections [26, 27, 67]. It has also been shown that LES prophages are active as free phage in the sputum of CF patients [28]. However, it is clear that LES isolates can vary in their complement of GIs and prophages, and also that some GIs and prophages were more often absent or divergent than others. For example, carriage of LES prophage 5 was restricted to sub-sets of isolates from the same CF region (Liverpool). This might support the notion that the strain first evolved in Liverpool, given that prophage 5 was present in the earliest known isolate, and that loss of the unstable prophage occurred before spread, but we cannot rule out acquisition restricted to some isolates. Interestingly, absence of the most variable GI (GI-5) was also mostly associated with sub-sets of isolates from this same region. In contrast, absence of GI-2 or GI-3 was rare, as was complete absence of the other LES prophages, implying that they contribute to the success of their host during chronic CF infections. In particular, genes associated with prophages 3 and 4 and GI GI-3 featured prominently amongst those genes associated with the LES in comparison to other *P. aeruginosa* strains, including in prophage 3, genes putatively encoding a HicA/HicB toxin–antitoxin system, implicated in the stringent response and persistence [68]. Previous studies support the notion that LES prophages contribute to the establishment of chronic lung infections [27], and our data support the idea that these accessory regions are making an important contribution to the success of the LES. It is worth noting that the isolate PHELES19, from an unusual source (blood), was also unusual in that it lacked GI-3 and most of the LES prophages. Generally, isolates that clustered closely according to core genome SNP phylogeny tended to share the same complement of GIs and prophages, suggesting that instability has occurred during transmission and spread of the LES lineage, but not so rapidly that closely related isolates do not broadly share the same accessory genome content.

### LES adaptations

We identified a number of loss-of-function mutations previously associated with the LES. For example, *fleR* mutations, associated with loss of motility, a characteristic of many *P. aeruginosa* isolates from CF infections [69], and a defining characteristic of the LES [29], were identified in all but one of the UK isolates but not in the isolates from Canada.

Although adaptations to the CF lung occurring by mutation have been widely reported [70], we also found evidence for adaptation via the loss of large genomic regions. We identified a large region, including genes for extracellular polysaccharide (Psl), a siderophore (pyoverdine) and a type VI secretion system, that was deleted from the genomes of 15 of the LES isolates (Fig. S4). The boundaries of these deletions differed between isolates, and additional, different deletions were present in some isolates, indicating the occurrence of independent deletion events and a tendency towards genome reduction during chronic infection. Biofilm formation is considered to be an important strategy for *P. aeruginosa* during chronic lung infections, and the bacterium can produce three different exopolysaccharides that contribute to this process, namely Psl, Pel and alginate [71]. Alginate has been strongly implicated in *P. aeruginosa* CF infections because of the observation that many isolates express a mucoid phenotype due to over-production of alginate because of mutations, especially in *mucA* [72, 73]. Although the contributions of Pel and Psl during CF infections are less well understood, it has been shown that a LES isolate favours expression of *pel* genes over *psl* genes in a murine model of chronic lung infection [74]. Mutations in the pyoverdine biosynthesis genes are a common adaptation in CF, and *P. aeruginosa* adapts to favour iron acquisition from haemoglobin rather than via siderophores [75, 76]. Hence the loss of genes encoding Psl or pyoverdine would be consistent with previous analysis of *P. aeruginosa* adaptions. Since the type VI secretion system is a weapon employed by bacteria to attack competitors, loss of this system in the highly competitive multi-species environment of the CF lung is harder to understand. However, it may be that in a biofilm lifestyle within the structurally heterogeneous environment of the CF lung, over time *P. aeruginosa* can establish itself in niches where there is no requirement for these weapons.

We also observed many independent mutations in genes previously implicated in adaptive responses during *P. aeruginosa* chronic lung infections [70]. Some of these were loss-of-function mutations. Mutations leading to mucoidy (*mucA*) or impairment of quorum sensing (*lasR*) are amongst the most common reported adaptations in *P. aeruginosa* chronic lung infections [70, 77]. Some of these were loss-of-function mutations. We also observed common antimicrobial resistance-associated mutations, including *pmrB* mutations, which have been associated with adaptation to the lung in a murine chronic infection model [78].

Inactivation of *oprD* in *P. aeruginosa* results in carbapenemase resistance, a growing problem with *P. aeruginosa* [79]. Decreased expression of *oprD* in LES400 and LES431 compared with PAO1 has been shown to correlate with an increase in minimum inhibitory concentrations (MICs) for meropenem [80]. Disruptive mutations in the PAO1 *mltD* homologue (PA1812) have been associated with increased beta-lactam antibiotic MICs [81] and in *P. aeruginosa* PA14 disruptive mutations in the *glpT* gene result in fosfomycin resistance [82]. Disruption of *mpl* homologues in *P. aeruginosa* have additionally been associated with piperacillin and ceftazidime resistance due to an accumulation of immature cell wall components and up-regulation of AmpC expression [83].

The loss of the MexAB-OprM efflux pump, due to severe mutations in any of the genes in the *mexAB-oprM* operon, has also been observed in non-CF bronchiectasis chronic lung infections [77]. This is harder to explain in terms of an adaptation leading to antibiotic resistance. The efflux pump has also been implicated in virulence, and it may be this alternative role that is more important in the CF lung adaptation [84].

Although we have identified mutations via analysis of the genomic data, we do not here present phenotypic data to demonstrate linkage between the genetic variations and specific phenotypic adaptations.

## Conclusions

Most CF patients acquire their *P. aeruginosa* from environmental sources; transmissible (epidemic) strains capable of spreading by transmission between patients are therefore unusual. Here we have demonstrated that isolates identified as the LES belong to a distinct lineage when compared to other *P. aeruginosa* strains and separate by core genome SNP phylogeny on the basis of country of isolation (UK or Canada). In addition to transmissibility, in both nations, the LES has been associated with greater patient morbidity [12, 85]. In CF, *P. aeruginosa* causes long-term chronic infections, and during the course of such infections *P. aeruginosa* populations not only acquire adaptive mutations, but also evolve to be heterogeneous [30, 70]. For our isolate collection, we cannot know how far down this adaptive process the *P. aer *uginosa population sampled has travelled at the point the isolate was obtained. Hence, attempts to reconstruct a global LES evolutionary history based upon substitution rates would not yield meaningful insights. However, we observed that the oldest known isolates (LESB58, from 1988) falls between the two major clades (associated with the UK or Canada), and that there are variations between the clades in terms of signature mutations and the carriage of accessory genes. These findings suggest (a) that the LES lineage established itself separately in the UK and Canada some considerable time ago and (b) that the special characteristics of the lineage in terms of greater competitiveness, the ability to spread and associations with increased morbidity, cannot easily be assigned to shared mutations or accessory gene content.

## Supplementary Data

Supplementary material 1

Supplementary material 2
