# Supplementary material for: Transmission, adaptation and geographical spread of the Pseudomonas aeruginosa Liverpool epidemic strain
Source: Microb Genom. 2021 Mar 15;7(3):mgen000511. doi: 10.1099/mgen.0.000511 (PMC8190615; doi:10.1099/mgen.0.000511)
Supplement: Supplementary material 1 [file mgen-7-0511-s001.pdf]

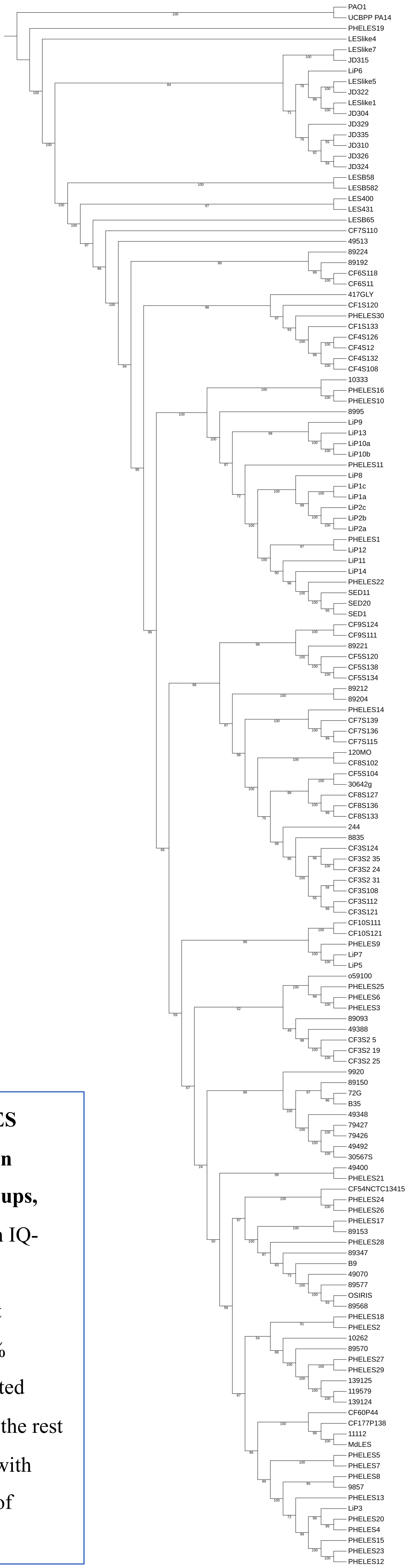

Fig S1. Phylogenetic approximation of the LES genomes included in this study and two known *Pseudomonas aeruginosa* phylogenetic outgroups, PAO1 and PA14. The tree was constructed with IQ-TREE(43) maximum likelihood, 1000 ultra-fast bootstrap supports from a relaxed core fragment supermatrix (500bp fragments present in >=95% genomes) with 14,730 informative sites and rooted between known outgroups PAO1 and PA14 and the rest of the tree. The tree was visualised by iTol(47) with colour strip indicating PHE designated regions of isolation or Canada

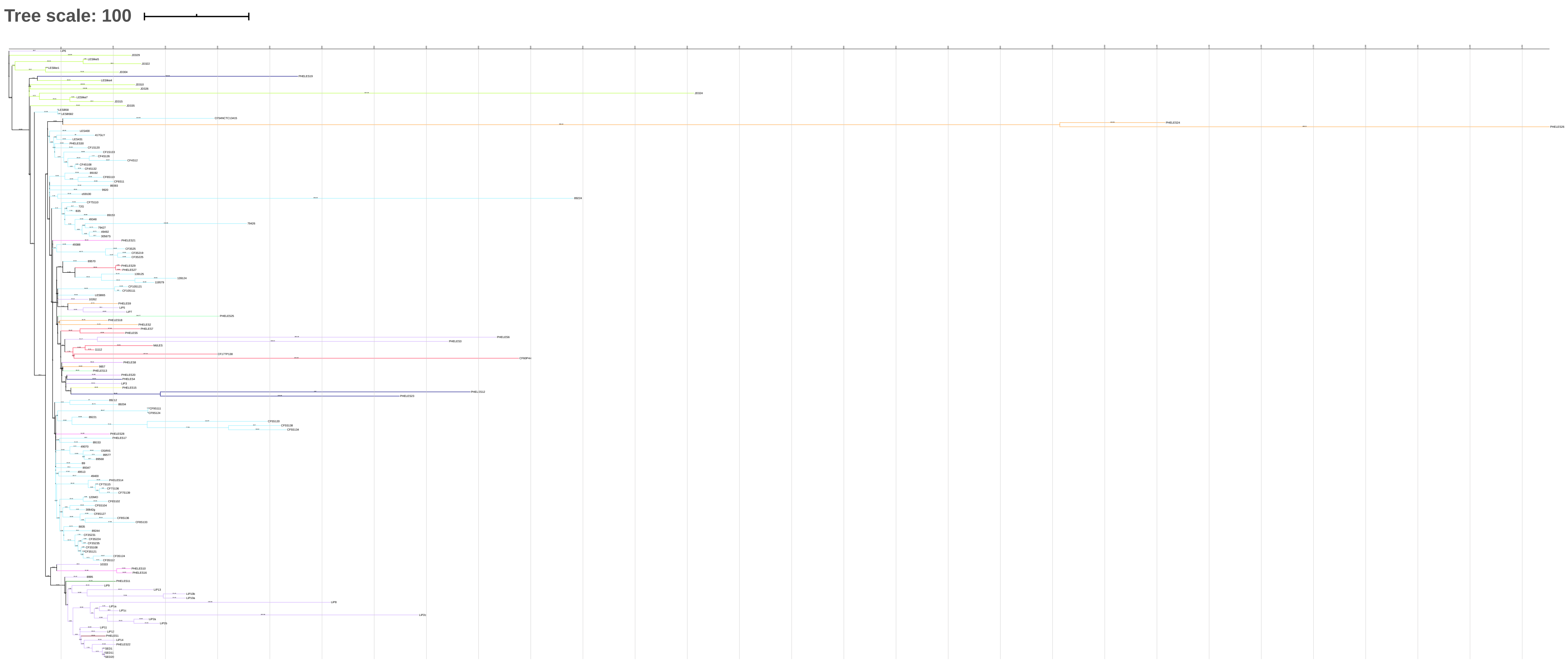

Fig S2. Phylogenetic tree inferred from core SNPs with LESB58 genomic islands and prophages masked with branch lengths shown. Recombinant sites determined by ClonalFrameML were excluded. The GTR+F+G4 substitution model was selected by IQ-TREE and there were 2,947 informative sites. The tree was visualised by iTol (47) with colour strip and branch colours indicating PHE designated regions of isolation or Canada. Branch lengths shown in SNPs.

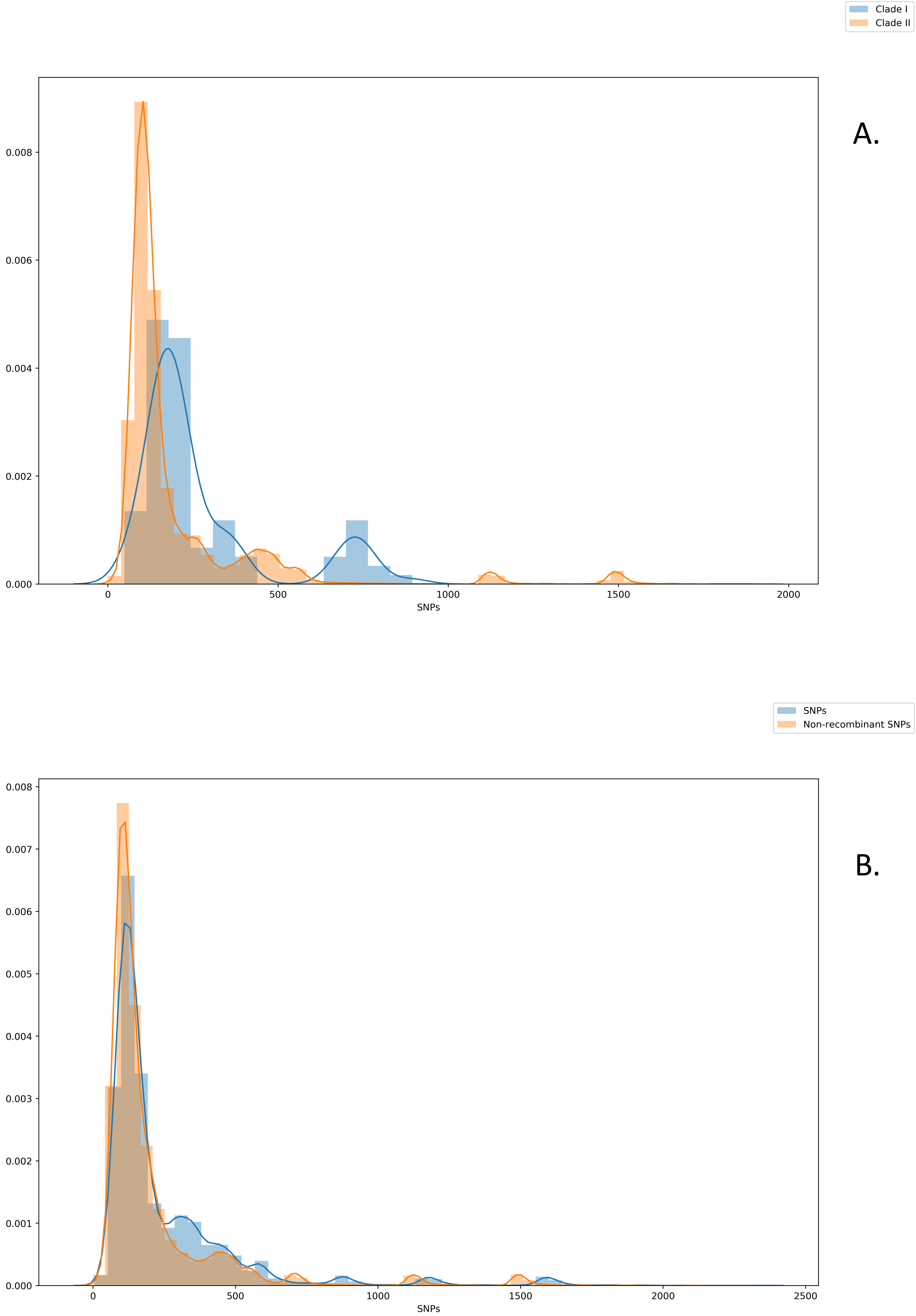

Fig S3. LES non-redundant pairwise SNP distance histograms

(A) Histogram of non-recombinant SNPs within clade I (primarily Canadian) and clade II (primarily UK) genomes exclusive pairs**.** (B) Histogram of exclusive pairwise SNP distances for SNPs for all genome pairs with and without recombinant sites removed.

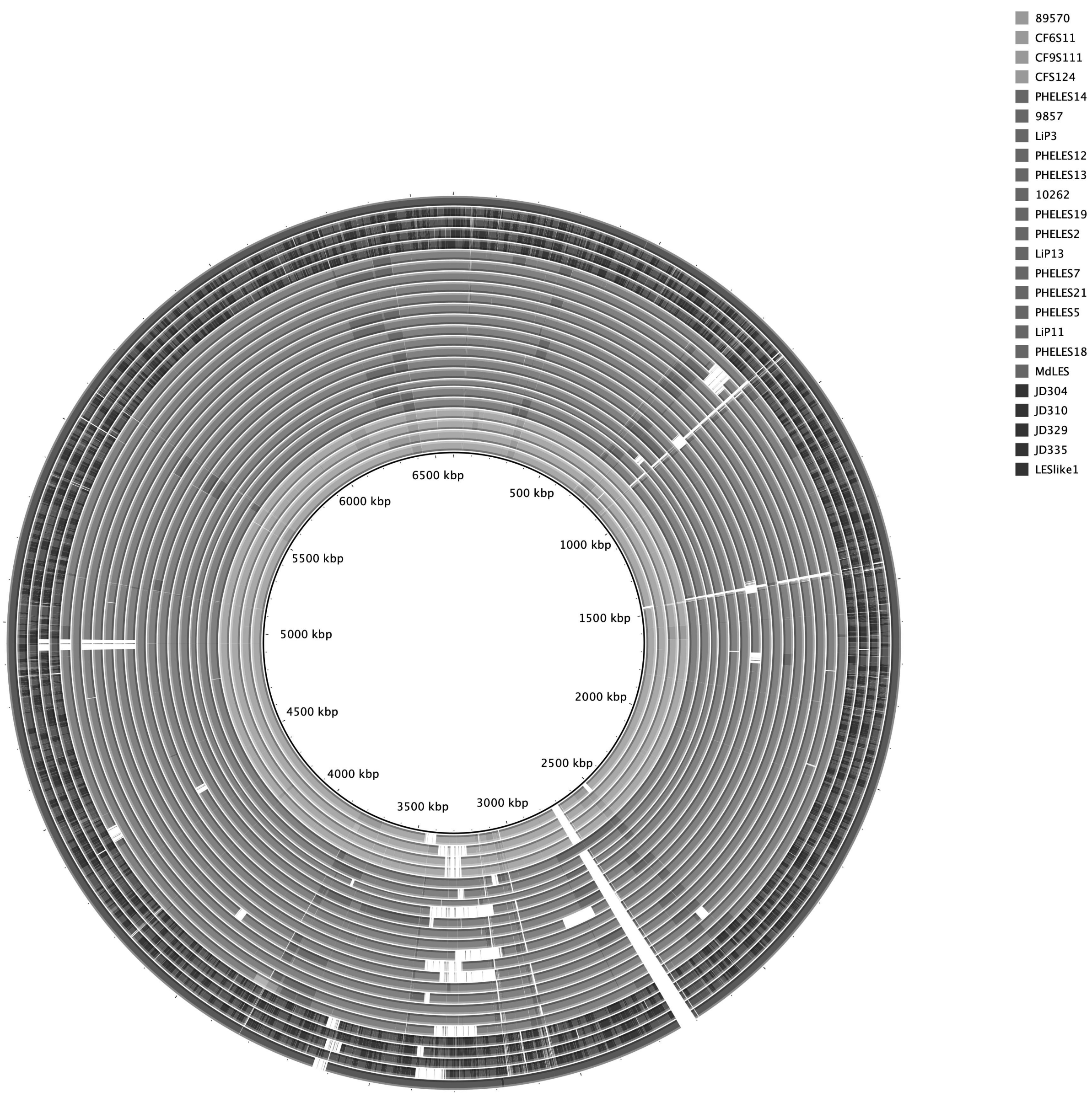

Fig S4. Deletions in genomes of LES strains.

Deletions in genomes of LES strains. The genomes of all deletion-containing strains were aligned with that of LESB58. Pale grey, isolates from Liverpool; medium grey, isolates from elsewhere in the United Kingdom; dark grey, isolates from Canada. Isolates are listed sequentially from the innermost circle. The locations of deletions are listed in Table S5. The image was generated using BRIG.
